# Developing Innolysins Against *Campylobacter jejuni* Using a Novel Prophage Receptor-Binding Protein

**DOI:** 10.3389/fmicb.2021.619028

**Published:** 2021-02-01

**Authors:** Athina Zampara, Martine C. Holst Sørensen, Yilmaz Emre Gencay, Dennis Grimon, Sebastian Hougaard Kristiansen, Lallana Skaarup Jørgensen, Josephine Rejgaard Kristensen, Yves Briers, Anne Elsser-Gravesen, Lone Brøndsted

**Affiliations:** ^1^Department of Veterinary and Animal Sciences, University of Copenhagen, Copenhagen, Denmark; ^2^Department of Biotechnology, Ghent University, Ghent, Belgium; ^3^ISI Food Protection ApS, Aarhus, Denmark

**Keywords:** *Campylobacter*, prophage binding, endolysin, Innolysin, antibacterials, food safety

## Abstract

*Campylobacter* contaminated poultry remains the major cause of foodborne gastroenteritis worldwide, calling for novel antibacterials. We previously developed the concept of Innolysin composed of an endolysin fused to a phage receptor binding protein (RBP) and provided the proof-of-concept that Innolysins exert bactericidal activity against *Escherichia coli*. Here, we have expanded the Innolysin concept to target *Campylobacter jejuni*. As no *C. jejuni* phage RBP had been identified so far, we first showed that the H-fiber originating from a CJIE1-like prophage of *C. jejuni* CAMSA2147 functions as a novel RBP. By fusing this H-fiber to phage T5 endolysin, we constructed Innolysins targeting *C. jejuni* (Innolysins Cj). Innolysin Cj1 exerts antibacterial activity against diverse *C. jejuni* strains after *in vitro* exposure for 45 min at 20°C, reaching up to 1.30 ± 0.21 log reduction in CAMSA2147 cell counts. Screening of a library of Innolysins Cj composed of distinct endolysins for growth inhibition, allowed us to select Innolysin Cj5 as an additional promising antibacterial candidate. Application of either Innolysin Cj1 or Innolysin Cj5 on chicken skin refrigerated to 5°C and contaminated with *C. jejuni* CAMSA2147 led to 1.63 ± 0.46 and 1.18 ± 0.10 log reduction of cells, respectively, confirming that Innolysins Cj can kill *C. jejuni in situ*. The receptor of Innolysins Cj remains to be identified, however, the RBP component (H-fiber) recognizes a novel receptor compared to lytic phages binding to capsular polysaccharide or flagella. Identification of other unexplored *Campylobacter* phage RBPs may further increase the repertoire of new Innolysins Cj targeting distinct receptors and working as antibacterials against *Campylobacter.*

## Introduction

*Campylobacter jejuni* is the major cause of foodborne gastroenteritis worldwide, leading to more than 245,000 human cases annually only in Europe, and is thus associated with a significant economic burden [European Food Safety Authority (EFSA), 2015]. *C. jejuni* colonizes the poultry intestine to high numbers and despite numerous efforts, sustainable solutions for controlling this pathogen is yet not available. The use of lytic phages for specific biocontrol of *C. jejuni* in primary poultry production has shown promising results, but is also hampered by phage resistance development (Loc Carrillo et al., 2005; Holst Sørensen et al., 2012; Fischer et al., 2013; Hammerl et al., 2014; Richards et al., 2019). So far, lytic phages of *C. jejuni* have been shown to recognize the capsular polysaccharides or the flagella (Sørensen et al., 2015) and application of phage cocktails targeting both receptors have led to less than 1 log reduction in bacterial counts on chicken skin (Zampara et al., 2017). Therefore, alternative antibacterial agents are needed to eliminate this pathogen. One such approach may be the design and use of phage-derived enzymes instead of replicating phages.

Endolysins are phage-encoded enzymes that degrade the peptidoglycan leading to osmotic imbalance and cell lysis. Endolysins have been successfully used as alternative antibacterials against diverse pathogenic bacteria, displaying low probability of resistance development (Nelson et al., 2012). However, their antibacterial activity is generally limited toward Gram-positive bacteria, as Gram-negative bacteria possess an outer membrane that hinders access of endolysins to the peptidoglycan layer (Fernández-Ruiz et al., 2018; Gutiérrez and Briers, 2021). Over the last decade, research has been focused on developing approaches to enable endolysins to overcome the outer membrane barrier of Gram-negative bacteria. Fusion of endolysins with the binding domains of bacteriocins is one of such approaches, allowing the hybrid endolysins to overcome the outer membrane barrier, putatively through the target outer membrane proteins (Lukacik et al., 2012; Yan et al., 2017; Heselpoth et al., 2019). Alternatively, endolysins fused with polycationic and amphipathic peptides appear to interfere with the outer membrane integrity. These artificial endolysins, known as Artilysins^®^, are under commercial development and with reported antibacterial activities against *Pseudomonas aeruginosa*, *Acinetobacter baumannii*, and colistin-resistant *E. coli* (Briers et al., 2014; Defraine et al., 2016; Schirmeier et al., 2018). Recently, we provided a proof of concept for the use of a phage receptor binding protein (RBP), enabling the fused endolysin to exert antibacterial activity and kill *E. coli* (Zampara et al., 2020). In our previous work, we constructed hundreds of RBP-endolysin hybrids (Innolysins) by fusing 24 different endolysins with Pb5 (phage T5 RBP) in different configurations. Screening of these Innolysin variants for antibacterial activity identified Innolysin Ec21, which was able to reduce *E. coli* resistant to third-generation cephalosporins by 3.31 ± 0.53 log. The antibacterial activity of Innolysins Ec was proved to be dependent on the Pb5 cognate receptor, FhuA (Zampara et al., 2020). Thus, the phage RBP provides specificity to the fused endolysin of the Innolysin Ec. Moreover, discovery of novel phage RBPs may allow development of Innolysins against other Gram-negative bacteria.

The identification of *Campylobacter* phage RBPs is challenged by the small number of in-depth characterized *Campylobacter* phages and publicly available genomes. Although a putative RBP (Gp047) of phage NCTC12673 was proposed and shown to bind to acetamidino-modified pseudaminic acid residues of the flagella, it was later reported unlikely to be an RBP, as it could not be detected in the mature virion (Javed et al., 2015). Thus, up to date no RBPs of lytic *C. jejuni* phages have been identified. However, putative RBPs of *Campylobacter* prophages may be used as a source of RBPs. The prophage elements of *C. jejuni* were discovered when the genome sequence of the chicken isolate *C. jejuni* RM1221 was compared to the reference genome of *C. jejuni* NCTC11168 and revealed three *C. jejuni* integrated elements (CJIEs) (Parkhill et al., 2000; Fouts et al., 2005). The first element known as CMLP1 or CJIE1was shown to be a Mu-like phage, which encodes several proteins similar to phage Mu, including transposase homologs, as well as the five-base (TATGC) direct repeats flanking the element (Fouts et al., 2005; Clark and Ng, 2008). Unfortunately, isolation of free phage particles has been repeatedly unsuccessful and so far, it is not understood how CJIE1-like prophages recognize their host and their RBPs have not been identified. Sequence analysis of CJIE1-like prophages of different strains showed several insertion and deletions (Clark and Ng, 2008; Clark, 2011) and also identified a region of 372 nucleotides with high variation in a gene suggested to encode the tail fiber protein H (Clark and Ng, 2008).

Here, we aimed to design Innolysins targeting *Campylobacter* (Innolysin Cj) by combining the specificity of an RBP protein with the antibacterial activity of endolysins. To do so, we first confirmed the function of a putative RBP found in CJIE1-like prophage in CAMSA2147 and then fused it to different endolysins for construction of Innolysins Cj. Two of the constructed Innolysins were able to kill *C. jejuni in vitro* and *in situ* on artificially contaminated chicken skin. Our work provides novel insights in designing alternative antibacterials with targeted killing against *Campylobacter*.

## Materials and Methods

### Bacterial Strains, Growth Conditions, and Media

Bacterial strains used in this study are listed in Table 1 and Table 2. C. *jejuni* strains were routinely grown on Blood Agar Base II (Oxoid) supplemented with 5% calf blood (BA) at 42°C under microaerobic conditions (6% CO_2_, 6% O_2_, 84.5% N_2_, and 3.5% H_2_). Luria-Bertani broth (LB) and LB agar (LA) (Difco) were used for growing *E. coli*, while Tryptic Soy Broth (TSB) and Tryptic Soy Agar (TSA) were used for *P. aeruginosa*. When needed 100 μg/ml of kanamycin and 50 μg/ml of chloramphenicol were used for selection of *E. coli* transformants or 15 μg/ml gentamicin for selection of *P. aeruginosa* transformants.

**TABLE 1:** Bacterial strains.

	Description	References
*P. aeruginosa* strains		
PAO1	Clinical wound isolate; R2-pyocin producer	Williams et al., 2008
PAO1 Δ*prf15*	*P. aeruginosa* PAO1 lacking the R2-pyocin tail fiber due to an in-frame deletion of codons 11–301 of *prf15*	Williams et al., 2008
PEG02	PAO1 Δ*prf15*/pM63, campycin + chaperone co-expression strain, Gm^r^ (15 μg/ml)	This study
PEG16	PAO1 Δ*prf15/*pM96, campycin expression strain, Gm^r^ (15 μg/ml)	This study
*E. coli* strains		
BL21-CodonPlus (DE3)-RIL	Contains a ColE1-compatible, pACYC-based plasmid containing extra copies of the argU, ileY, and leuW tRNA genes and was used as a protein expression strain, Cam^r^ (50 μg/ml)	Agilent Technologies
InCj1	BL21-CodonPlus (DE3)-RIL/pAZInCj1, *Innolysin Cj1* + *chaperone* co-expression strain, Kan^r^ (100 μg/ml), Cam^r^ (50 μg/ml)	This study
InCj2	BL21-CodonPlus (DE3)-RIL + pAZInCj2, *Innolysin Cj2* + *chaperone* co*-*expression strain, Kan^r^ (100 μg/ml), Cam^r^ (50 μg/ml)	This study
InCj5	BL21-CodonPlus (DE3)-RIL + pAZInCj5, *Innolysin Cj5* + *chaperone* co-expression strain, Kan^r^ (100 μg/ml), Cam^r^ (50 μg/ml)	This study
AZE1	BL21-CodonPlus (DE3)-RIL/pAZE1, *T5 endolysin* expression strain, Kan^r^ (100 μg/ml), Cam^r^ (50 μg/ml)	This study
AZE5	BL21-CodonPlus (DE3)-RIL/pAZE5, *Salmonella* phage Shivani peptidase expression strain, Kan^r^ (100 μg/ml), Cam^r^ (50 μg/ml)	This study
B195	BL21-CodonPlus (DE3)-RIL/CRYS3, C-terminal of *H-fiber* + *chaperone* co-expression strain, Kan^r^ (100 μg/ml), Cam^r^ (50 μg/ml)	This study
*C. jejuni* strains		
NCTC11168	A preferred strain for studying phage-host interactions at the molecular level	National collection of type cultures
NCTC11168 Δ*kpsM*Δ*motA*	A capsular and non-motile flagellated NCTC11168 mutant	Baldvinsson et al., 2014

**TABLE 2:** *Campylobacter jejuni* strains used for testing antibacterial activity of Innolysins Cj.

*C. jejuni strain*	ENA accession number^a^	MLST^b^	MLST CC^c^	Origin
CAMSA2147	GCA_003095855	21	ST-21	Chicken
CAMSA2068	GCA_003095815	22	ST-22	Chicken
CAMSA2054	Not published	354	ST-354	Chicken
CAMSA2135	GCA_003095715	45	ST-45	Chicken
CAMSA2118	Not published	1,326	ST-45	Chicken
CAMSA2020	Not published	48	ST-48	Chicken
CAMSA2136	Not published	692	ST-692	Chicken
CAMSA2043	Not published	441	−	Chicken
CAMSA2025	Not published	4,748	−	Chicken
CAMSA2019	GCA_003095725	4,811	−	Chicken

**^*a*^Accession number based on European Nucleotide Archive (ENA). ^*b*^Multilocus sequence typing. ^*c*^Multilocus sequence typing clonal complexes.**

### Bioinformatic Analysis

To predict the protein function and the domains of CAMSA2147 prophage H-fiber (WP_002878910.1) and the downstream putative chaperone (WP_002878909.1), we mined CJIE1-like prophage sequences in our collection using CJIE1 of *C. jejuni* RM1221 (NC_003917.7) as query. Blast similarity allowed determining a CJIE1-like prophage in the genome assembly of CAMSA2147. Then, gene syntheny was inspected manually, in comparison to phage Mu and phage P2. Respective domains were determined using InterProScan (Jones et al., 2014) and HMMER (Finn et al., 2011).

### Plasmid Constructions

#### Construction of Plasmids for Campycin Expression

Multiple steps were conducted to construct plasmids pM63 and pM96 composed of the coding sequence for the N-terminal domain of R2-pyocin Prf15 fused with the coding sequence of the C-terminal domain of the CAMSA2147 CJEI-like prophage H-fiber (WP_002878910.1) with or without the downstream gene for the putative chaperone (WP_002878909.1). Initially, the coding sequence of the N-terminal domain of R2-pyocin Prf15 (amino acids 1–164) was cloned into pUCPtac plasmid (Williams et al., 2008) giving rise to pM50 (Table 3). Therefore, *P. aeruginosa* PAO1 was used as a template for the amplification of the fragment using R2N primers (Supplementary Table S1), which add *Sal*I and *Hin*dIII restriction sites at the ends of the fragment. Then, the pUCPtac plasmid was linearized via inverse PCR using specific primers (Supplementary Table S1) and ligation was conducted with T4 DNA ligase. This plasmid (pM50) (Table 3) was further used as backbone for creating the fusions using In-Fusion^®^ cloning—Takara Bio. Therefore, the fragment comprising the coding sequence of the C-terminus of the H-fiber (amino acids 151–343) and the downstream chaperone gene was amplified from CAMSA2147 (GCA_003095855) by specific primers that add overhangs identical to the distal ends of the linearized pM50 (Supplementary Table S1). Inverse PCR was conducted for the linearization of pM50. Recombination of the fragment with the ends of the linearized pM50 was performed according to the In-Fusion^®^ HD cloning kit user manual, resulting in plasmids pM63 and pM96, respectively. Stellar competent cells were used for transformation and transformants were selected on LB agar plates in the presence of 15 μg/ml gentamicin. *P. aeruginosa* PAO1 Δ*prf15* were transformed with the extracted plasmids as described previously (Choi et al., 2006).

**TABLE 3:** Plasmids used or constructed.

Plasmid	Construction	Used for	References
pUCPtac	Modified pUCP30T (Gm^r^ broad-host-range plasmid, Accession no: U33752) containing *lacl*^q^ tac MCS^a^	Cloning vector	Williams et al., 2008
pET-28a (+)	N-His, N-Thrombin, C-His, Kan^r^ (100 μg/ml)	Expression vector	Novagen
pM50	pUCPtac:N-terminus of *prf15* of R2-pyocin lacking the binding domain, Gm^r^ (15 μg/ml)	Engineering R2-pyocins	This study
pM63	pM50:C-terminus of *H-fiber gene* + *chaperone gene* of CAMSA2147 CJIE1, Gm^r^ (15 μg/ml)	Campycin co-expression with chaperone	This study
pM96	pM50:C-terminus of *H-fiber gene* of CAMSA2147 CJIE1, Gm^r^ (15 μg/ml)	Campycin expression	This study
pAZE1	pET-28a (+):*T5lys* from phage T5, Kan^r^ (100 μg/ml)	T5 endolysin expression	This study
pAZE5	pVTD: *Salmonella phage Shivani peptidase gene*, Kan^r^ (100 μg/ml)	*Salmonella* phage Shivani peptidase expression	This study
pCRYS3	pET-28a (+):C-terminus of *H-fiber gene* + *chaperone gene* of CAMSA2147 CJIE1, Kan^r^ (100 μg/ml)	H-fiber expression	This study
pAZInCj1	pET-28a (+):*Innolysin Cj1* + *chaperone gene* of CAMSA2147 CJIE1, Kan^r^ (100 μg/ml)	Innolysin Cj1 expression	This study
pAZInCj2	pET-28a (+):*Innolysin Cj2* + *chaperone gene* of CAMSA2147 CJIE1, Kan^r^ (100 μg/ml)	Innolysin Cj2 expression	This study
pAZInCj5	pVTD:*Innolysion Cj5* + *chaperone* of CAMSA2147 CJIE1, Kan^r^ (100 μg/ml)	Innolysin Cj5 expression	This study

**^*a*^MCS, multiple cloning site.**

#### Construction of Plasmids for Innolysin Cj Expression

Coding sequences for Innolysins Cj1 and Innolysin Cj2 were chemically synthesized by GeneCust (Luxembourg) and cloned into pET-28a (+) using the *Nde*I and *Xho*I restriction sites (Table 3). Plasmid pAZInCj1 expressing Innolysin Cj1 was designed by fusing the coding sequence of H-fiber protein of CJIE1-like prophage originating from CAMSA2147 (WP_002878910.1) to the C-terminus of phage T5 endolysin coding sequence (YP_006868.1). A linker encoding 14 amino acids (Linker 2, Supplementary Table S2) was used to join both coding sequences. The same configuration was used for plasmid pAZInCj2 expressing Innolysin Cj2, but instead of the whole H-fiber, the C-terminal part of H-fiber coding sequence was used, excluding the N-terminal DUF3751 domain. Both plasmids pAZInCj1 and pAZInCj2 also contain the flanking downstream gene of CAMSA2147, the putative fiber chaperone (WP_002878909.1). In-Fusion^®^ cloning was used to clone either the T5 endolysin or the C-terminus (amino acids 151 to 343) of the H-fiber (WP_002878910.1) along with the downstream chaperone gene (WP_002878909.1) into peT28a (+), giving rise to the control plasmids pAZE1 or pCRYS3, respectively (Table 3). Amplification of these genes was performed with specific primers (Supplementary Table S1) and using phage T5 (Leibniz Institute DSMZ) or *C. jejuni* CAMSA2147 DNA as a template, respectively. The vector pET28a (+) was linearized using *Nde*I and *Xho*I restriction enzymes and insertion was performed based on the In-Fusion^®^ HD cloning manual. Plasmids were obtained after transformation of *E. coli* Stellar competent cells and selection on LB agar with kanamycin (100 μg/ml). Subsequently, all plasmids were used to transform *E. coli* BL21-CodonPlus(DE3)-RIL (Agilent Technologies) by plating on LB agar plates in the presence of kanamycin (100 μg/ml) and chloramphenicol (50 μg/ml). All constructs were sequenced by Sanger sequencing.

### Expression of Campycins and R2-Pyocins

Expression of campycin and native R2-pyocin, as a negative control, was conducted as described previously (Williams et al., 2008). Briefly, *P. aeruginosa* PAO1 expressing the native R2-pyocin or campycin [i.e, a tail fiber deficient R2-pyocin derivative (Δ*prf15*) substituted *in trans* with the H-fiber along with or without the chaperone (PEG02 or PEG16)] were grown overnight in TSB at 37°C. The overnight cultures were 100-fold diluted in G medium (Ikeda and Egami, 1969), which was supplemented with 15 μg/ml gentamicin in the case of PEG02 and PEG16, and were incubated at 37°C until reaching an OD_600_ of 0.25. R2-pyocins were induced by 3 μg/ml mitomycin, while isopropyl-beta-D-thiogalactopyranoside (0.25 mM) was added to PEG02 and PEG16 for induction of the expression of the engineered H-fiber in *trans*. Cultures were incubated for 2.5 h at the same conditions, followed by treatment with DNase I (Invitrogen) at a final concentration of 5 U/ml and further incubated for another 30 min at the same conditions. To remove cell debris, cultures were centrifuged at 18,000 *g* at 4°C for 1 h and the supernatants were harvested and treated with saturated ammonium sulfate solution at a final concentration of 1.6 M, while stirring on ice. Suspensions were incubated overnight at 4°C with shaking and the pellets containing the precipitated campycins/pyocins were harvested after centrifugation at 4°C and 18,000 *g* for 1 h. The precipitates were resuspended in 1/10th of the start volume with ice cold TN50 buffer (50 mM NaCl and 10 mM Tris-HCl adjusted to pH 7.5) and buffer exchange was performed with TN50 buffer by using Amicon Ultra-centrifugal Filter Units with Ultracel-15 with 30 kDa membrane cutoff (Merck Millipore). Concentration of campycins/pyocins was measured with a Qubit 2.0 fluorometer and adjusted to 0.15 mg/ml with TN50 buffer.

### Determination of the Antibacterial Activity of Campycins and R2-Pyocins

To assess the ability of campycins to kill *Campylobacter*, a semi-quantitative assay was initially conducted by spotting 5 μl onto *Campylobacter* bacterial lawns. Preparation of *C. jejuni* CAMSA2147 lawns was performed as previously described (Gencay et al., 2017). Briefly, bacterial cells were harvested from the plate with calcium Brain- Heart Infusion Broth (cBHI) and adjusted to an OD_600_ of 0.35. These suspensions were further incubated for 4 h at 42°C under microaerobic conditions (6% CO_2_, 6% O_2_, 84.5% N_2_ and 3.5% H_2_) and 500 μl were mixed with 5 ml of molten NZCYM overlay agar [NZCYM broth (Sigma) with 0.6% agar (Sigma)]. The mixture was then poured on NZCYM basal agar [with 1.2% agar (Sigma)] plates, containing 10 μg/ml vancomycin [Sigma]. Plates were dried for 45 min in the flow hood and prepared campycins/pyocins were spotted on top, followed by overnight incubation at 42°C under microaerobic conditions. The antibacterial activity was assessed by the formation of a distinct clear zone as a result of cell killing on the lawns. The native R2-pyocin or fiber mutant derivative (Δ*prf15*) were also spotted onto *Campylobacter* bacterial lawns as negative controls.

Antibacterial activity of campycin was also assessed by measuring the bacterial log reduction in colony forming units (cfu). *C. jejuni* was harvested from the plate with cBHI and adjusted to an OD_600_ of 0.35 followed by tenfold dilution until the final concentration reached 10^6^ cfu/ml. Then, 9 ml of the culture was mixed with either 1 ml of campycin at the final concentration of 0.015 mg/ml or 1 ml of TN50 buffer, followed by incubation for 3 h at 42°C under microaerobic conditions. After incubation, proper dilutions were made, and cells were plated on BA plates. Incubation of plates was followed for 24 h at 42°C under microaerobic conditions and cfu/ml was calculated based on biological triplicates.

### Screening Innolysins Cj for Muralytic Activity

To screen Innolysins Cj for muralytic activity, proteins were expressed in *E. coli* BL21-CodonPlus-(DE3)-RIL cells, as previously described (Zampara et al., 2020). Briefly, freshly transformed colonies were re-suspended in 500 μl of auto-induction medium [93% ZY medium, 0.05% 2M MgSO_4_, 2% 50 × 5052 (0.5% glycerol, 0.05% glucose, and 0.2% α-lactose in 20 ml water), 5% 20 × NPS (50 mM Na_2_HPO_4_, 50 mM KH_2_PO_4_, and 25 mM (NH_4_)_2_SO_4_ to 50 mL of water)]. Incubation was followed at 37°C for 5 h at 900 rpm and switched to 16°C for 40 h at 900 rpm. Samples were centrifuged (3,200 × *g*, 30 min, 4°C) and cell pellets were lysed by exposure to chloroform vapor for 2 h. Five hundred microliters of 20 mM HEPES-NaOH (pH 7.4) with 1 U DNase I was used for resuspension of cell lysates and samples were incubated for 1 h at 30°C. Cell suspensions were centrifuged (3,200 × *g* for 30 min, at 4°C) and the soluble fractions of lysates present in the supernatants (cleared lysates) were used for the muralytic assay as previously described (Lavigne et al., 2004; Briers et al., 2014). Peptidoglycan of *P. aeruginosa* PAO1 was used as substrate, since it displays the same chemotype (A 1γ) as *E. coli* and *C. jejuni* (Schleifer and Kandler, 1972). To permeabilize *P. aeruginosa*, pellets of exponentially growing cells (OD_600_ = 0.6) were harvested by centrifugation (3,200 × g, 30 min, 4°C) and resuspended in chloroform-saturated 0.05 M Tris-HCl (pH 7.7) buffer. Gentle shaking for 45 min allowed (outer) membrane permeabilization, followed by two washing steps with phosphate-buffered saline (PBS, pH 7.4). The turbidity of the outer membrane-permeabilized cells was adjusted to OD_600_ = 1.5 and 270 μl was used as a substrate, while 30 μl of Innolysins Cj cleared lysates was applied on the top of the substrate in triplicate. Cleared lysates of cells carrying the empty vector pET-28a (+) or expressing phage T5 endolysin were used as a negative and a positive control, respectively. Turbidity was measured spectrophotometrically at 650 nm every 30 s for 1 h using a Microplate Reader 680 system (Bio-Rad) and a standardized method was used to calculate the activities (Briers et al., 2007).

### Expression and Purification of Innolysins Cj

To test the antibacterial activity, Innolysins Cj were expressed by growing strains InCj1, InCj2, InCj5, and controls strains AZE1 and B195 in 1 L auto-induction medium. Cultures were incubated for 4 h at 37°C and switched to 15°C for 18 h at 120 rpm. Cells were centrifuged (8,000 × *g*, 10 min, 4°C) and pellets were further resuspended in 10 ml of lysis buffer (20 mM NaH_2_PO_4_-NaOH, 0.5 M NaCl, 50 mM imidazole, pH 7.4). Cells were lysed by sonication (Bandelin Sonopul HD 2070 homogenizer) with 10 bursts of 30 s (amplitude of 50%) and 30 s intervals. Cell lysates were filtered twice with 0.22 μm pore size filters and protein purification was conducted by using His GraviTrap^TM^ gravity flow columns (GE Healthcare). The lysis buffer was also used for the wash step, while elution step was conducted by using 6 ml of 20 mM NaH_2_PO_4_-NaOH, 0.5 M NaCl, 500 mM imidazole, pH 7.4. Amicon Ultra-15 Centrifugal Filter Units with Ultracel-10 membrane cutoff (Merck Millipore) were used for exchange of the elution buffer with 20 mM HEPES-NaOH (pH 7.4). Sodium dodecyl sulfate- polyacrylamide gel electrophoresis (SDS-PAGE) was used to determine purity of samples (Supplementary Figure S1) and protein concentration was measured with a Qubit 2.0 fluorometer.

### *In vitro* Antibacterial Activity of Purified Innolysins Cj

The killing efficiency of Innolysins Cj was tested on different *Campylobacter* strains (Table 2). *C. jejuni* strains were cultured on Mueller Hinton (MH) agar plates for 24 h at 42°C under microaerobic conditions (6% CO_2_, 6% O_2_, 84.5% N_2_, and 3.5% H_2_). MH broth was used to harvest and to exponentially grow the cells until they reach an optical density OD_600_ of 0.25. The cell suspensions were further diluted with MH down to a concentration of 10^5^ cfu/ml. 100 μl of the diluted samples were mixed with either 100 μl of purified Innolysin at the final concentration of 1 mg/ml or 100 μl of 20 mM HEPES-NaOH (pH 7.4) instead, for negative controls. After incubation for 45 min at 20°C, proper dilutions were made and plated on BA agar plates. Overnight incubation was followed at 42°C under microaerobic conditions and cfu were counted. Cell concentrations (cfu/ml) were calculated and cell reductions were estimated as a difference of the average logarithmic cell concentrations of the cultures treated with Innolysin compared to the negative control. The experiment was conducted in biological triplicate.

### Preparation of Innolysin Cj Library

To prepare the Innolysin Cj library, we used the previously described VersaTile technique (Gerstmans et al., 2020). Initially, a semi-random combinatorial library with each variant composed of four tiles was constructed: one H-fiber tile at position 1, seven linker tiles at position 2, 25 enzymatic activity domain (EAD) tiles at position 3 and one hexahis-tag tile at position 4. The library has a complexity of 175 (= 1^∗^7^∗^25^∗^1) possible variants. The EAD and linker tiles were previously constructed (Supplementary Tables S2, S3) and were readily available as plasmid stocks (Gerstmans et al., 2020). The H-fiber tile (RBP8) was created according to the protocol for tile preparation described in Gerstmans et al. (2020). Briefly, the RBP coding sequence was first amplified using tailed primers (Supplementary Table S1) and purified using gel extraction (GeneJet Gel Extraction Kit, Thermo Fisher scientific). Subsequently, the fragment was cloned in the entry vector (pVTE) using 2U T4 DNA ligase (Thermo Fisher Scientific), 5U sapI (Thermo Fisher Scientific), 26 nM pVTE, 46 nM fragment and ligase buffer (Thermo Fisher Scientific). 5 μL of the resulting reaction mixture was used to transform chemically competent *E. coli* TOP10 cells, followed by selective plating on LB agar supplemented with 5% (w/v) sucrose and ampicillin (100 μg/mL). T he sequence of each tile was verified by Sanger sequencing (LGC genomics). Subsequently, the VersaTile assembly mixture was made taking 1 μL of 46 nM entry vector containing the corresponding tile or a mixture of tiles for each position, 26 nM pVTD, 2U T4 DNA ligase (Thermo Fisher Scientific), 5U *Bsa*I (Thermo Fisher Scientific), and ligase buffer (Thermo Fisher Scientific). This mixture was incubated for 50 cycles alternating between 37°C (5 min) and 16°C (5 min), followed by heat inactivation at 50°C (5 min) and 80°C (5 min). The resulting reaction mixture was used to transform competent *E. coli* BL21(DE3)-RIL cells and the transformed cells were subsequently plated on LB 1.5% agar plates supplemented with kanamycin (50 μg/mL) and 5% (w/v) sucrose. VersaTile technique was also used for cloning the gene encoding *Salmonella* phage Shivani peptidase (YP_009194685) into pVTD vector (Supplementary Figure S2), using the tile carrying the responsible gene at position 1 and the tile carrying the hexahis-tag at position 2. The resulting reaction mixture was used to transform competent *E. coli* BL21(DE3)-RIL as mentioned above.

### Screening of Innolysin Cj Library for Growth Inhibition

After transformation, 96 clones were randomly selected and screened for growth inhibition. The library variants were expressed using auto-induction medium in a 96 deep-well plate and the cleared lysates were harvested as described in the screening of Innolysins Cj for muralytic activity. The ability of the Innolysins Cj to inhibit the cell growth was tested on CAMSA2147 similar to previous work (Zampara et al., 2020). Briefly, CAMSA2147 was cultured on MH agar plates for 24 h at 42°C under microaerobic conditions (6% CO_2_, 6% O_2_, 84.5% N_2_, and 3.5% H_2_) and cells were harvested with 2×MH broth and adjusted to 0.05 optical density OD_600_. 50 μl of the diluted cultures were mixed with 50 μl of the soluble lysate fraction of cells expressing each Innolysin. CAMSA2147 mixed with 20 mM HEPES-NaOH (pH 7.4) buffer was used as negative control. Mixed samples were incubated for 24 h at 42°C under microaerobic conditions (6% CO_2_, 6% O_2_, 84.5% N_2_, and 3.5% H_2_). Lack of growth was assessed as reduced turbidity of cultures compared to the negative control by measuring the optical density spectrophotometrically at 600 nm. This high-throughput screening was performed with technical triplicates.

### *In situ* Antibacterial Activity of Innolysins Cj

The antibacterial activity of either Innolysin Cj1 or Innolysin Cj5 was tested against CAMSA2147 on artificially contaminated chicken skin supplied from a Danish slaughterhouse. Frozen chicken skins were defrosted on the day of the experiments and were aseptically cut into 3×4 cm (12 cm^2^) pieces. Each of the chicken pieces was treated with 20 μl of the strain containing in total 10^4^ cfu that were evenly spread over the surface and immediately thereafter exposed to 50 μl of each Innolysin (200 μg) or 50 μl of 20 mM HEPES-NaOH (pH 7.4) as a negative control. Samples were exposed for 45 min under modified packaging conditions (30% CO_2_, 70% N_2_) at 5°C. After exposure, skin pieces were put in plastic bags and weighted. Cells were harvested by using 10 ml of 20 mM HEPES-NaOH (pH 7.4) buffer and by shaking the samples in the stomacher for 1 min. Harvested solutions were serially tenfold diluted in 20 mM HEPES-NaOH (pH 7.4) and plated on Campy Rapid plates that are selective for *Campylobacter*. Incubation of plates was followed for 48 h under microaerobic conditions at 42°C. *C. jejuni* colonies were counted and compared with the number of recoverable bacteria of the control samples. Average log reduction of bacterial cells per gr was estimated based on biological triplicate.

### Statistical Analysis

Statistical analysis of the results was conducted by using GraphPad Prism 7 software (Version 7.0d). The Paired-Samples *t*-test with 95% confidence interval percentage was used to test the significance of logarithmic bacterial reduction of cells treated with either campycin or Innolysins Cj compared to the negative controls (cells treated with TN50 buffer or 20 mM HEPES-NaOH (pH 7.4), respectively). To test whether the muralytic activities of cleared lysates of cells expressing Innolysins were significant compared to the negative control [muralytic activity of cleared lysate of cells carrying empty vector, pET28a (+)], the Unpaired-Samples *t*-test was used with 95% confidence interval based on biological triplicates.

## Results

### Identification of Campylobacter Phage RBP for Construction of Innolysins Cj

**FIGURE 1:**
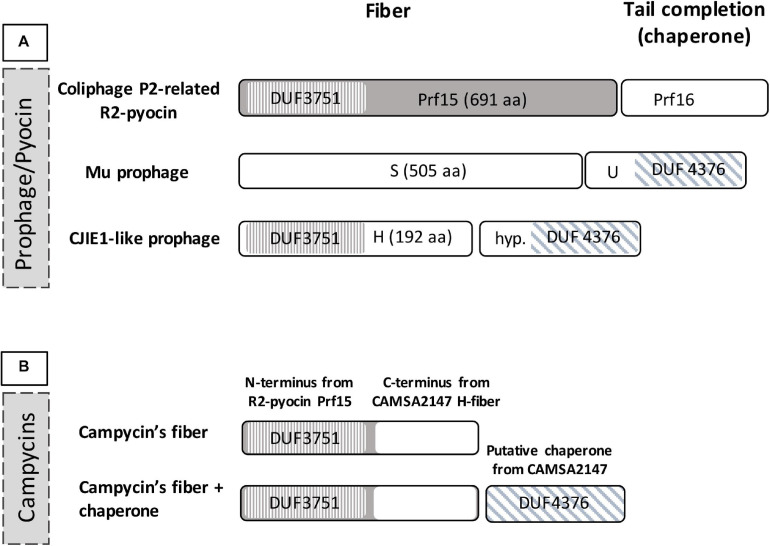
Domains of the tail fiber region of CJIE1-like prophages and construction of campycins. **(A)** Domains of H-fiber *C. jejuni* prophage CJIE1-like in comparison to prophage Mu and R2-pyocin. The H-fiber of CJIE1-like prophages harbors an N-terminal DUF3751 domain similar to the *P. aeruginosa* PAO1 R2-pyocin tail fiber encoded by *prf15* (Williams et al., 2008). The gene downstream of the H-fiber gene is predicted to encode a DUF4376 domain as found in the U gene that functions as a chaperone for the Mu phage tail fiber (Haggard-Ljungquist et al., 1992). **(B)** For construction of campycin, the DUF3751 domain encoded by *P. aeruginosa* PAO1 R2-pyocin was fused with the C-terminus of the CAMSA2147 H-fiber.

In order to construct Innolysins against *Campylobacter*, we first aimed to identify the RBP of the CJIE1-like prophage in *C. jejuni* CAMSA2147. Since mutations often accumulate in RBPs due to adaptations to variations in host receptors (Nobrega et al., 2018), the observed variability of the H-fiber of CJIE1-like prophages (Clark and Ng, 2008) may indicate that it functions as the RBP of the prophage. *C. jejuni* CAMSA2147 also carries an CJIE1-like prophage encoding a H-fiber. Bioinformatic analysis showed that the N-terminus of this H-fiber harbors a DUF3751 domain analogous to the N-terminal domains of the coliphage P2 protein H and the Prf tail fiber of R2-pyocin in *P. aeruginosa* PAO1 (Figure 1A). This tail fiber domain functions as an anchor, allowing the tail fiber to attach to the R2-pyocin (Williams et al., 2008). Moreover, the gene downstream of the H-fiber gene has a DUF4376 domain similar to the U gene located downstream of the tail fiber gene of phage Mu. The U gene encodes a chaperone that is required for the functionality of the phage Mu RBP (Haggard-Ljungquist et al., 1992; North et al., 2019). Therefore, our bioinformatic analysis showed that the H-fiber shares common features with RBPs of other phages, suggesting that it may function as an RBP for the CJIE1-like prophages.

### The Tail Fiber Protein H of Campylobacter CJIE1-Like Prophage Functions as an RBP

To demonstrate binding of the H-fiber to *Campylobacter*, we exploited the principle of tail fiber switching of the R2-pyocin. The R2-pyocin is a bacteriocin morphologically similar to the tail complex of simple myoviruses such as coliphage P2 or Mu (Nakayama et al., 2000; Leiman and Shneider, 2012). Its tail fibers bind to the bacterial receptors, followed by contraction of the tail and pore formation in the cell membrane, thus dissipating the bacterial membrane potential leading to cell lysis (Michel-Briand and Baysse, 2002; Ge et al., 2020). Thus, to show that the H-fiber functions as an RBP, we engineered the R2-pyocin by exchanging the receptor binding domain of the tail fiber of native R2-pyocin with the C-terminus of the H-fiber gene of CJIE1-like prophage from *C. jejuni* CAMSA2147 (Figure 1B). We named this engineered pyocin campycin, because the presence of the C-terminus of the H-fiber should redirect the bactericidal effect to *Campylobacter*. We further co-expressed the campycin with the putative chaperone in case it may be needed for the functionality of the H-fiber (Figure 1B). All constructs were transformed to a *P. aeruginosa* PAO1 derivative carrying all other components of the R2-pyocin but lacking the tail fiber due to a deletion of gene *prf15*. The substituting tail fiber was thus expressed *in trans*, allowing formation of the campycins.

**FIGURE 2:**
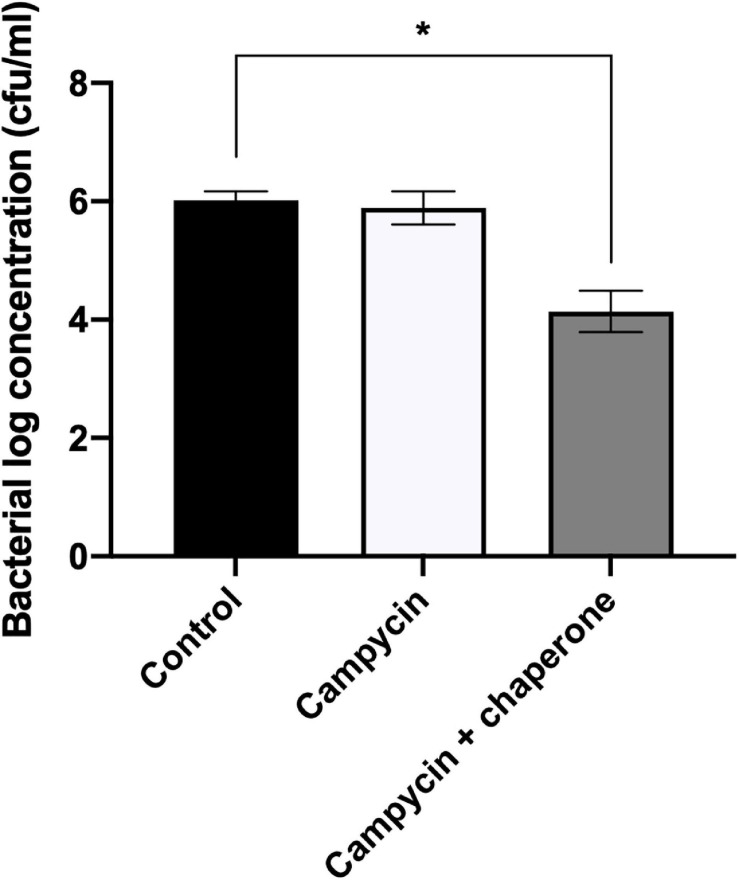
Antibacterial activity of campycin against *Campylobacter* CAMSA2147. Campycin was expressed with or without chaperone and mixed with CAMSA2147. TN50 buffer was used instead of campycins as a negative control. Samples were incubated for 3 h in microaerobic conditions at 37°C. Colony forming units per milliliter (cfu/ml) were counted. Experiments were performed in triplicates. Error bars represent the standard deviations of the mean. *Significant reduction at *P* < 0.05.

We expressed and spotted campycin on CAMSA2147 bacterial lawns. The appearance of lysis zones on bacterial lawns indicate the ability of campycin to bind and kill the cells (Table 4). Interestingly, the campycin only caused lysis of CAMSA2147 bacterial lawns when it was co-expressed with the putative chaperone. In contrast, the campycin alone, the native R2-pyocin or the native R2-pyocin lacking the tail fiber (Prf15) were not able to lyse CAMSA2147. These results prove that the H-fiber of the CJIE1-like prophage is able to bind to *C. jejuni*, suggesting that it functions as an RBP and that the downstream putative chaperone is required for its functionality. To further confirm that the H-fiber of campycin binds to and kills *C. jejuni*, reduction of CAMSA2147 colony forming units (cfu) was determined after treatment with campycin expressed with or without the chaperone. Indeed, an average 1.88 log reduction of cells was shown when CAMSA2147 was treated with the campycin co-expressed with the chaperone (Figure 2), whereas the campycin alone did not lead to significant reduction of cells compared to the control (cells treated with TN50 buffer). Currently described receptors for lytic *Campylobacter* phages include the capsule or the flagellum (Sørensen et al., 2015). To investigate whether any of these structures functions as the receptor for the H-fiber of CJIE1-like prophage, native R2-pyocin and campycin were tested for cell lysis on bacterial lawns of *C. jejuni* NCTC11168 lacking both the capsule and flagella (NCTC11168 Δ*kpsM*Δ*motA*) and the wildtype strain as a control. A clear lysis zone was observed on NCTC11168 Δ*kpsM*Δ*motA* similar to the wildtype (Table 4), indicating that the campycin recognizes a receptor different from the capsule and flagella. Overall, the H-fiber derived from the CJIE1-like prophage of CAMSA2147 functions as a novel *Campylobacter* phage RBP that requires the downstream located chaperone to be functional.

**TABLE 4:** Formation of clear lysis zones on lawns of different *Campylobacter* strains.

Bacterial strains	Native R2-pyocin	R2-pyocin lacking fiber^a^	Campycin	Campycin + chaperone
*C. jejuni* CAMSA2147	−	−	−	**+**
*C. jejuni* NCTC11168	−	−	−	**+**
*C. jejuni* NCTC11168 Δ*kpsM*Δ*motA*^b^	−	−	−	**+**

**^*a*^P. aeruginosa PAO1 R2-pyocin lacking the tail fiber due to deletion of responsible gene prf15. ^*b*^Mutant of NCTC11168 lacking both the capsule and a motile flagella.**

### Campylobacter Targeting Innolysins Remain Muralytic Active

**FIGURE 3:**
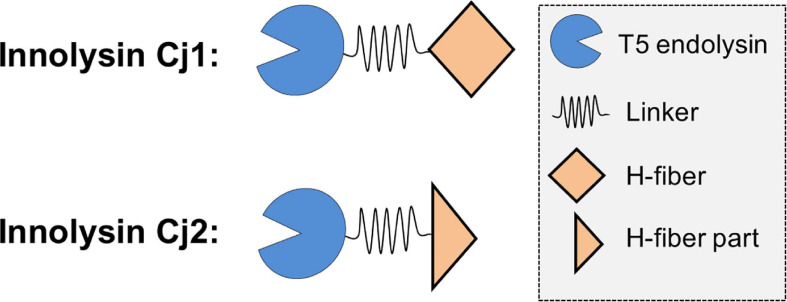
Construction of Innolysins Cj. Innolysin Cj1 was constructed by fusing the C-terminus of phage T5 endolysin to the tail fiber protein H of *Campylobacter* CAMSA2147 CJIE1-like prophage. These two domains were fused by using a linker in between composed of 14 amino acids (GAGAGAGAGAGAGA). The same configuration was used for Innolysin Cj2 but instead of the whole H-fiber, the C-terminal part of the H-fiber was used, excluding the N-terminal DUF3751 domain.

We utilized the newly discovered RBP to target Innolysins against *C. jejuni* (Innolysins Cj). Therefore, we fused the H-fiber to phage T5 endolysin and anticipated that binding of the H-fiber to *C. jejuni* allows the fused endolysin to overcome the outer membrane barrier and to exert antibacterial activity. Innolyins Cj were designed with the same configuration as our previously most efficient Innolysin targeting *E. coli* (InEc21). InEc21 was composed of an N-terminal endolysin and a C-terminal RBP, fused with a flexible linker (Zampara et al., 2020). Thus, Innolysins Cj1 was composed of the T5 endolysin in the N-terminus fused by a long flexible linker with the H-fiber in the C-terminus. The same configuration was used for Innolysin Cj2, but only using the C-terminal part of the H-fiber without the DUF3751 domain as an RBP component (Figure 3). Furthermore, both Innolysins Cj were co-expressed with the chaperone of CAMSA2147 CJIE1-like prophage to ensure functionality of the H-fiber.

**FIGURE 4:**
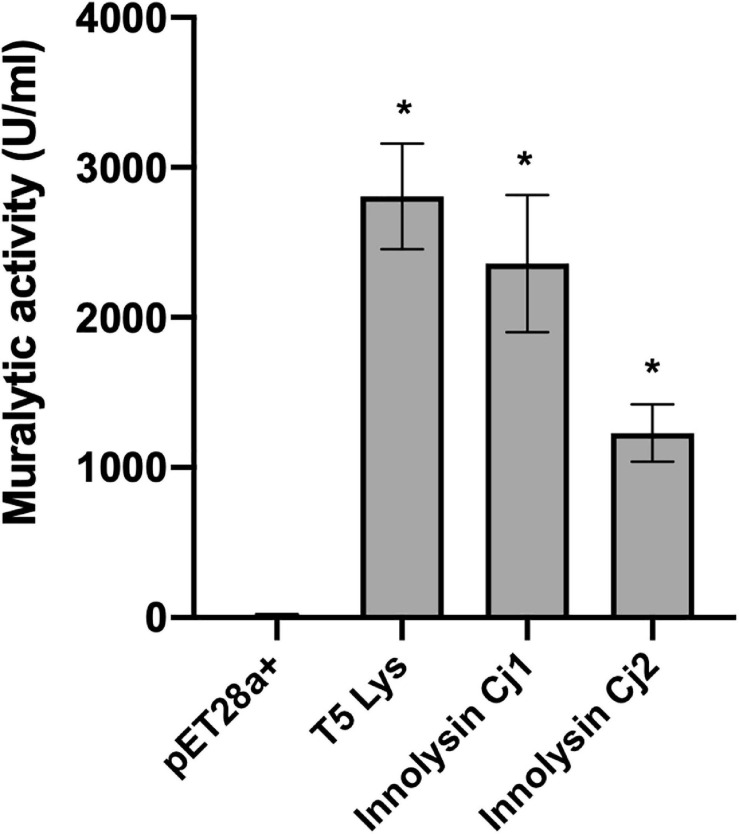
Muralytic activity of Innolysins Cj. Enzymatic activities of Innolysins Cj1 and Cj2 were tested on outer membrane-permeabilized *P. aeruginosa* substrate in triplicate. Cleared lysates of cells expressing Innolysins Cj were used for the assay. Cleared lysates of cells expressing either the phage T5 endolysin (T5 Lys) or carrying the empty vector pET-28a (+) were used as positive and negative controls, respectively. Enzymatic activities were depicted as units per milliliter (U/ml) without normalization for product expression yield. Error bars represent the standard deviations of the mean. *Significant muralytic activity at *P* < 0.05.

To verify that the T5 endolysin remains enzymatically active within the hybrid proteins, we tested the ability of Innolysins Cj1 and Cj2 to degrade peptidoglycan. The enzymatic activity of cleared lysates of expressed Innolysins Cj was determined using outer membrane permeabilized *P. aeruginosa* cells, which serve as a reference substrate for peptidoglycan degrading activity (Briers et al., 2007). Innolysin Cj1 and Innolysin Cj2 displayed muralytic activities of 2,360 and 1,229 U/ml, respectively, while T5 endolysin alone has a muralytic activity of 2,807 U/ml (Figure 4). Yet, precise comparisons are not possible because cleared lysates were used, thus the observed activities are dependent on both product yield and specific activity. Overall, our results indicate that both Innolysins are able to exert enzymatic activity and degrade the peptidoglycan.

### Innolysins Cj Exert Antibacterial Activity Against Various *Campylobacter* Strains

**FIGURE 5:**
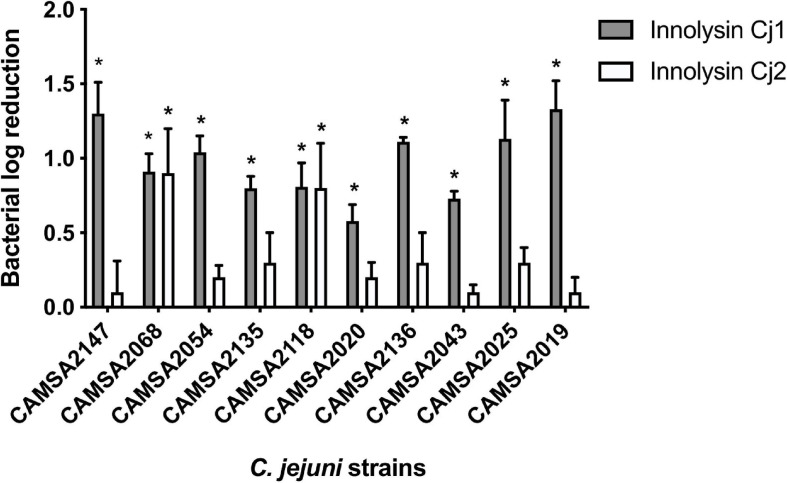
Antibacterial spectrum of Innolysins Cj against *C. jejuni* strains. The bacterial log reduction of different *C. jejuni* strains was determined after application of 1 mg/ml purified Innolysin Cj1 or Innolysin Cj2 and exposed for 45 min at 20°C. Average logarithmic reductions of treated cells were measured compared to the cells treated with 20 mM HEPES-NaOH (pH 7.4) (Innolysins buffer solution). The *C. jejuni* strains were derived from the Statens Serum Institute (SSI) collection isolated from poultry and pork in Denmark, showing different multilocus sequence typing (MLST) and different MLST clonal complexes (CC) (Table 2). The average logarithmic reduction was calculated based on three independent experiments. Error bars represent the standard deviations of the mean. *Significant reduction at *P* < 0.05.

To evaluate whether Innolysins Cj1 and Cj2 exert antibacterial activity against *Campylobacter*, we treated *Campylobacter* CAMSA2147 with 1 mg/ml of purified Innolysin Cj1 or Innolysin Cj2 for 45 min at 20°C. Innolysins Cj1 led to 1.30 ± 0.21 log reduction of cell counts, in contrast to Innolysin Cj2 that did not lead to significant log reduction (0.10 ± 0.21) (Figure 5). Furthermore, no significant log reduction was detected when cells were treated with 1 mg/ml of either the H-fiber (0.05 ± 0.03) or the T5 endolysin (0.16 ± 0.15) alone. These results indicate that the whole H-fiber may be a more potent RBP component compared to the C-terminal part of H-fiber. To determine the antibacterial spectrum of Innolysins Cj, the antibacterial activity of each Innolysin was tested against nine *Campylobacter* strains belonging to distinct multilocus sequence types (MLST) (Djordjevic et al., 2007; Table 2). All strains were sensitive to Innolysin Cj1, leading to a maximum of 1.33 ± 0.19 log reduction in cell numbers of CAMSA2019. In contrast, Innolysin Cj2 only exerted antibacterial activity against two strains (CAMSA2068 and CAMSA2118), reaching to 0.90 ± 0.30 log reduction of CAMSA2068 cells (Figure 5). Thus, it appears that Innolysin Cj1 is a better antibacterial candidate with a wider antibacterial spectrum compared to Innolysins Cj2. Overall, we showed that the H-fiber can be used as an RBP component of Innolysins to specifically kill diverse *Campylobacter* strains.

### Construction and Screening of an Innolysin Cj Library Yields an Additional Antibacterial Candidate

To possibly enhance the antibacterial activity of Innolysins Cj, we designed a library of novel Innolysins Cj by changing the configuration and using other EADs and linkers. For the construction of the library, we used the VersaTile technique (Gerstmans et al., 2020), which is a dedicated method for the combinatorial assembly of modular proteins. Specifically, the library variants were designed as such that the whole H-fiber is located now in the N-terminus and fused by one of seven possible linkers to one of 25 different EADs of phage lytic enzymes in the C-terminus. All variants also have a C-terminal hexahis-tag for purification purposes. The library has thus a final complexity of 175 possible variants. To identify the Innolysin Cj candidate with improved antibacterial activity, we used a high-throughput screening method based on bacterial growth inhibition. Specifically, 96 variants were randomly picked after transformation and screened by mixing CAMSA2147 cells with cleared cell lysates expressing an Innolysins Cj variant, followed by monitoring of the optical density of the cultures during 24 h. CAMSA2147 cells mixed with 20 mM HEPES-NaOH (pH 7.4) buffer served as a negative control, and led to an OD_600_ of 0.77 ± 0.04. The highest growth inhibitory effect was observed for Innolysin Cj5 that reduced growth to an OD_600_ of 0.33 ± 0.05. Sequencing revealed that Innolysin Cj5 was composed of an EAD with a putative endopeptidase activity from *Salmonella* phage Shivani (Supplementary Table S3) fused with a 13 amino-acid long rigid linker (LINK7, Supplementary Table S2) to the N-terminal H-fiber. To determine the antibacterial activity of Innolysin Cj5, we further purified Innolysin Cj5 and tested the antibacterial activity against CAMSA2147. Exposure to Innolysin Cj5 led to 1.16 ± 0.04 log reduction of cells, which was in a similar range as Innolysin Cj1. In contrast, application of *Salmonella* phage Shivani endolysin alone did not significantly reduce CAMSA2147 cells under the same conditions (0.28 ± 0.02 log reduction). Thus, changing the configuration of Innolysins Cj did not enhance the antibacterial activity, but the configuration is flexible as for both opposite configurations more than 1 log reduction of *Campylobacter* cells was identified.

### Antibacterial Activity of Innolysin Cj1 and Cj5 on Chicken Skin

To test whether Innolysins Cj could kill *Campylobacter* present on food products, we tested the antibacterial activity of either Innolysin Cj1 or Innolysin Cj5 against CAMSA2147 *in situ*. Chicken skin was inoculated with CAMSA2147 (10^4^ CFU) and treated immediately afterward with 200 μg of either Innolysin Cj1 or Innolysin Cj5. For negative controls, 20 mM HEPES-NaOH (pH 7.4) buffer was used instead. After 45 min of exposure of the samples under modified packaging conditions (30% CO_2_, 70% N_2_) at 5°C, the log reduction of cells per gr chicken skin was assessed. A 1.63 ± 0.46 and 1.18 ± 0.10 log reduction was shown when CAMSA2147 was treated with either Innolysin Cj1 or Innolysin Cj5, respectively, compared to the negative controls. These results are in accordance with our *in vitro* experiments and show that Innolysins Cj have the potential to be used as antibacterial agents against *Campylobacter* on food.

## Discussion

Phage-derived RBPs are broadly and elegantly being exploited for designing novel antibacterials due to the specificity that they provide when they bind to the receptors on the bacterial surface (Dams et al., 2019). For example, we recently used a phage RBP to enable endolysin to overcome the outer membrane barrier and to kill Gram-negative bacteria (Zampara et al., 2020). We constructed Innolysins Ec composed of an EAD fused with the phage T5 RBP, Pb5 to specifically kill *E. coli*. Screening of a large library of Innolysins Ec for antibacterial activity allowed us to select Innolysin Ec21, displaying bactericidal activity against both laboratory and antibiotic-resistant *E. coli* strains (Zampara et al., 2020). Here, we expanded upon the Innolysin concept by developing Innolysins to kill *C. jejuni*, which is responsible for more than 90% of cases of campylobacteriosis (Sheppard et al., 2009; Kaakoush et al., 2015).

Since no *C. jejuni* phage RBPs have been identified so far, we used bioinformatic analysis to predict that the H-fiber protein encoded by CJIE1-like prophages functions as an RBP. To demonstrate the function, we engineered R2-type pyocin to carry the C-terminus of the H-fiber derived from CAMSA2147 CJIE1-like prophage and showed that it is able to kill *C. jejuni* strains. Interestingly, the H-fiber requires co-expression of the downstream gene to function as an RBP. T he C-terminus of the coding sequence of this gene contains a DUF4376 domain that displays homology to the C-terminus of the *E. coli* Mu phage tail fiber assembly (Tfa) protein (Tfa_*Mu*_). Tfa proteins are composed of a variable N-terminal domain that binds to the C-terminal region of the tail fiber and a conserved C-terminus (DUF4376) that mediates assembly and multimerization of the fiber (North et al., 2019). Similarly, the C-terminus of the protein identified downstream of the H-fiber of CJIE1-like prophages may be involved in oligomerization and assembly of the fibers. Furthermore, Tfa_*Mu*_ is an intermolecular chaperone that remains bound to the fiber of phage Mu, however, the exact function remains to be clarified (Haggard-Ljungquist et al., 1992; North et al., 2019). In other phages like *E. coli* phage T2 and T6, the downstream genes encode proteins that bind to the tail fibers and regulate recognition of bacterial receptors (Riede et al., 1987). Therefore, the downstream gene may influence the functionality of the H-fiber of CJIE1-like prophage by tail fiber oligomerization and assembly and/or recognition of the receptor. While lytic *C. jejuni* phages have been shown to recognize either flagella or capsular polysaccharides as receptors (Sørensen et al., 2015), our data suggests that the H-fiber and thus CJIE1-like prophages recognize a previously not described receptor. Overall, we provide novel insight of phage-host interaction of CJIE1-like prophages by identifying the RBP and we provide a new component for redirecting the antibacterial activity of Innolysins to *Campylobacter*.

By using the H-fiber derived from CAMSA2147 as the RBP component, we constructed two Innolysins Cj to kill *Campylobacter jejuni*, by fusing it to the C-terminus of T5 endolysin with a flexible linker in between. Innolysin Cj1 composed of the whole H-fiber as RBP component killed all 10 *Campylobacter* strains tested, reaching to 1.30 ± 0.21 log reduction of CAMSA2147 cells. In contrast, Innolysin Cj2 composed of the C-terminal part of the H-fiber could only significantly kill two out of the 10 strains. We previously suggested that the mode of Innolysins action relies on the binding of Innolysins RBP component to the bacterial receptors. This binding brings the fused endolysin to close proximity to the cell surface, where the positive charge of the endolysin may interfere with the outer membrane stabilizing cations (Zampara et al., 2020). As a result, the outer membrane integrity is impaired (Smoluch et al., 2016), allowing the endolysin to access and thereby degrade the peptidoglycan. Thus, we expect that the whole H-fiber may bind with higher affinity to the receptor compared to the C-terminal part of H-fiber, resulting to a broader antibacterial activity of Innolysin Cj1 compared to Innolysin Cj2. Further engineering allowed us to construct a library of Innolysins with a different configuration compared to Innolysin Cj1 and Innolysin Cj2. Based on a high-throughput screening for growth inhibition, we selected Innolysin Cj5 as a potent antibacterial candidate, leading to 1.16 ± 0.04 log reduction of CAMSA2147 cells *in vitro*. Although we did not identify an Innolysin with increased antibacterial activity compared to Innolysin Cj1, the platform offers opportunities for developing additional Innolysins Cj as antibacterial candidates. This is a novel concept for developing agents to control *Campylobacter* by exploiting phage RBPs and muralytic enzymes.

Chicken skin may be contaminated with high levels of *C. jejuni* during multiple stages of processing (Hansson et al., 2015; Biesta-Peters et al., 2019; Iannetti et al., 2020). Therefore, we used chicken skin as a food model system to test the antibacterial activity of Innolysins Cj against *C. jejuni*. Application of either Innolysin Cj1 or Innolysin Cj5 under food storage conditions led to 1.63 ± 0.46 and 1.18 ± 0.10 log reduction per gram chicken skin, respectively. Although the killing efficiency of Innolysin Cj1 by 1.63 ± 0.46 log reduction appears moderate, it has been predicted that campylobacteriosis cases derived from consumption of contaminated chicken meals could be decreased 30 times by a 2 log reduction of *Campylobacter* numbers on the chicken carcasses (Rosenquist et al., 2003). Thus, the antibacterial activity of Innolysins Cj could have a substantial impact on food safety and thus the number of human disease cases. Application of phage cocktails *in situ* against C. *jejuni* NCTC12662 under similar set up on chicken skin did not exceed 1 log reduction (Zampara et al., 2017). Therefore, Innolysins appear more efficient antibacterial agents compared to such phage application. Furthermore, Innolysin Cj approach offers advantages compared to phage application, because phage resistance can emerge due to *Campylobacter* intracellular adaptive mechanisms, such as intragenomic rearrangement between Mu-like prophages and phase-variable restriction modification systems (Scott et al., 2007; Anjum et al., 2016). In the case of Innolysins, bacterial resistance is most likely limited to mutations of the receptor for Innolysin binding. This could subsequently result in a reduced virulence, depending on the nature of the bacterial receptor. Since Innolysin Cj1 is able to kill a diverse spectrum of *C. jejuni*, the H-fiber appears to bind to a conserved receptor. Furthermore, a combined application of Innolysins targeting different receptors on *Campylobacter* at the same time may reduce the risk of bacterial resistance, as mutation of multiple receptors would be required instead of one. Here we showed that engineering of R2-pyocins of *P. aeruginosa* is a useful platform for discovery of novel RBPs to be used for development of novel Innolysins Cj. Overall, we used a novel RBP originating from a prophage to develop Innolysins Cj and expand the reservoir of potent antibacterials against *Campylobacter.*

## Data Availability Statement

The original contributions presented in the study are included in the article/Supplementary Material, further inquiries can be directed to the corresponding author/s.

## Author Contributions

AZ and YG performed most of the experiments. SK, LJ, and JK also performed some experiments. LB, MS, AE-G, and AZ designed the experiments. YB and DG collaborated in the library preparation and evaluation of the results. AZ, YG, and LB wrote the manuscript. All authors critically reviewed the manuscript and approved the submitted version.

## Conflict of Interest

The authors declare that the research was conducted in the absence of any commercial or financial relationships that could be construed as a potential conflict of interest.
