# Supplementary material for: Developing Innolysins Against Campylobacter jejuni Using a Novel Prophage Receptor-Binding Protein
Source: Front Microbiol. 2021 Feb 1;12:619028. doi: 10.3389/fmicb.2021.619028 (PMC7882524; doi:10.3389/fmicb.2021.619028)
Supplement: Supplementary file 1 [file Table_1.DOCX]

Supplementary Material

# Supplementary Tables

## Supplementary Table 1: Primers used for amplification of fragments cloned into either pUCPtac, pM50, pET28a (+) and pVTE vectors. Underlined sequences are complementary to the ends of the linearized vectors.

| **pUCPtac** |  |
| --- | --- |
| pUCPtac Frw | ACAGAGCTCGGTACCTCGAGGGCACTGGCCGTCGTTTTACAAC |
| pUCPtac Rvs | TCGGAGTATTGGTCGTCATGAATACTGTTTCCTGTGTGAAATTGTTATC |
| R2N Frw | AAACAGTATTCATGACGACCAATACTCCGAAATACGGT |
| R2N Rvs | GGTACCGAGCTCTGTAAGCTTTTCCTTCACCCAGTCCTGAGTG |
| **pM50** |  |
| pM50 Fw | AGCTTACAGAGCTCGGTACCTCGAG |
| pM50 Rvs | AAGCTTTTCCTTCACCCAGTCCTGAGTG |
| Hfiber Frw | GGTGAAGGAAAAGCTTGCTATTAAAGTGGAACTCAATCTTAAAATTGATGC |
| Hfiber Rvs | CGAGCTCTGTAAGCTTTATCTAAGCTCCATAAAAGATACAATGCTTTTGC |
| Hfiber+chaperone Frw | GGTGAAGGAAAAGCTTGCTATTAAAGTGGAACTCAATCTTAAAATTGATGC |
| Hfiber+chaperone Rvs | CGAGCTCTGTAAGCTTCATTTTTCACTCTCCCAAACAATTAAATCAAGTTCT |
| **pET28a (+)** |  |
| Hfiber+chaperone Frw | CGCGCGGCAGCCATATGGCTATTAAAGTGGAACTCAATCTTAAAATTGATG |
| Hfiber+chaperone Rvs | GGTGGTGGTGCTCGATCATTTTTCACTCTCCCAAACAATTAAATCAAGTTC |
| T5lys Frw | CGCGCGGCAGCCATATG AGT TTT AAA TTT GGT |
| T5lys Rvs | GGTGGTGGTGCTCGAGTTTAAACTAGTTCGACATG |
| **pVTE** |  |
| RBP8 Frw | TGTGCTCTTCAAGAGGTCTCACCATGGCAAAAAGTGAATACTATACCATACTAAC |
| RBP8 Rvs | TATGCTCTTCCCTTGGTCTCTGCACCTCTAAGCTCCATAAAAGATACAATGC |

**1.2 Supplementary Table 2. Used linkers (LINK) for library preparation of Innolysins Cj.** All linkers where used on position 2, hereby fusing the receptor binding protein with the used enzymatic domains.

| **Code** | **Position** | **Description** | **Origin** | **Amino acid length** | **Amino acid composition** |
| --- | --- | --- | --- | --- | --- |
| LINK1 | 2 | Flexible short linker | *In silico* design | 6 | AGAGAG |
| LINK2 | 2 | Flexible median linker | *In silico* design | 14 | GAGAGAGAGAGAGA |
| LINK3 | 2 | Rigid short helix | *In silico* design | 9 | LSRFFHAEL |
| LINK4 | 2 | Rigid long helix | *In silico* design | 14 | VFNQRKEHKGYMLA |
| LINK5 | 2 | Rigid long coil | *In silico* design | 16 | IPQGRSHPVQPYPGAF |
| LINK6 | 2 | Rigid short coil | *In silico* design | 6 | PAVPPP |
| LINK7 | 2 | Rigid, optimal helix | *In silico* design | 13 | EAAAKEAAKEAAK |

**1.3 Supplementary Table 3. Used Enzymatic Activity Domains (EADs) for construction of a Innolysin Cj library.** In total 25 EADs were used on position 3, resulting in constructs with a C-terminal EAD.

| **Code** | **Description** | **Origin** | **Pfam** | **Accession number** |
| --- | --- | --- | --- | --- |
| EAD1 | *PsP3gp10* | *Salmonella enterica* phage PsP3 | Phage lysozyme CL0037 | NP_958065.1 |
| EAD2 | *P2gp09* | *Escherichia coli* phage P2 | Phage lysozyme CL0037 | NP_046765.1 |
| EAD3 | *K11gp3.5* | *Klebsiella pneumoniae* phage K11 | Amidase_2 N-acetylmuramoyl-L-alanine amidase | AAX62800.1 |
| EAD4 | *CR8gp3.5* | *Citrobacter* phage CR8 | Amidase_2 N-acetylmuramoyl-L-alanine amidase | YP_009004176.1 |
| EAD5 | *LUZ24gp67* | *Pseudomonas aeruginosa* phage LUZ24 | Lysozyme | YP_001671940.1 |
| EAD6 | *BcepC6Bgp22* | *Burkholderia cepacia* phage BcepC6B | Phage lysozyme CL0037 | YP_024942.1 |
| EAD7 | *LysEC8* | *Escherichia phage* phAPEC8 | Phage lysozyme CL0037 | YP_007348465 |
| EAD8 | *KZ144-EAD* | *Pseudomonas* phage phiKZ | Transglycosylase SLT domain CL0037 | NP_803710.1 |
| EAD9 | *EL188-EAD* | *Pseudomonas* phage EL188 | - | YP_418221 |
| EAD10 | *PVP-SE1gp146-EAD* | *Salmonella* phage PVP-SE1 | Chitinase class I CL0037 | YP_004893952 |
| EAD12 | *201Phi2-1gp229-EAD* | *Pseudomonas chlororaphis* phage 201phi2-1 | (DUF3380 Protein of unknown function) | YP_001956952.1 |
| EAD13 | *Acibel* | *Acinetobacter baumannii* phage vB_AbaP_Acibel007 | - | YP_009103259 |
| EAD14 | *vB_PsyM_KIL1gp019* | *Pseudomonas* phage vB_PsyM_KIL1 | DUF3380 Protein of unknown function | YP_009276009.1 |
| EAD15 | *XccLys* | *Xantohomonas campestris* pv. campestris phage | Unpublished sequence | - |
| EAD16 | *Shivanigp41* | *Salmonella* phage Shivani | Peptidase_M15_4 | YP_009194685 |
| EAD17 | *Vpept* | *Vibrio* phage VvAW1 | D-alanyl-D-alanine carboxypeptidase cl00813 | YP_007518361 |
| EAD18 | *KMVgp36C* | *Pseudomonas* phage phiKMV | Lysozyme | NP_877475.1 |
| EAD19 | *KZgp181* | *Pseudomonas* phage phiKZ | Transglycosylase SLT domain | AAL83082.1 |
| EAD20 | *BcepC6Bgp16* | *Burkholderia* virus BcepC6B | Transglycosylase SLT domain | YP_024936.1 |
| EAD21 | *OBPgp276* | *Pseudomonas fluorescens* phage OBP | Glyco_hydro_19 | YP_004958183.1 |
| EAD22 | *KP32gp15* | *Klebsiella pneumoniae* phage KP32 | Amidase_2 N-acetylmuramoyl-L-alanine amidase | YP_003347533.1 |
| EAD29 | *Ph2119* | *Thermus scotoductus* phage | N-acetylmuramoyl-L-alanine amidase | AHF20915.1 |
| EAD30 | *Ts2631* | *Thermus scotoductus* phage | - | AIM47292.1 |
| EAD31 | *LysC* | *Clostridium intestinale* phage | - | ERK30183.1 |
| EAD40 | *OBPgp149* | *Pseudomonas fluorescens* phage OBP | - | YP_004958056.1 |

## 2. Supplementary Figures

**Supplementary Figure 1. Sodium dodecyl sulfate- polyacrylamide gel electrophoresis (SDS-PAGE) images produced after purification of proteins.**

1. Ruler
2. Cleared lysate of cells expressing Innolysin Cj1 (before purification)
3. Innolysin Cj1 after purification (expected size 56 kDa)
4. Cleared lysate of cells expressing Innolysin Cj2 (before purification)
5. Innolysin Cj2 after purification (expected size 38 kDa)
6. Ruler
7. H-fiber part after purification (expected size 22 kDa)
8. T5 endolysin after purification (expected size 16 kDa)
9. Ruler
10. Crude lysate of cells expressing Innolysin Cj5 (before purification)
11. Cleared lysate of cells expressing Innolysin Cj5 (before purification)
12. Innolysin Cj5 after purification (expected size 56 kDa)
13. Cleared lysate of cells expressing *Salmonella* phage Shivani peptidase (before purification)
14. *Salmonella* phage Shivani peptidase after purification (expected size 16 kDa)
15. Ruler


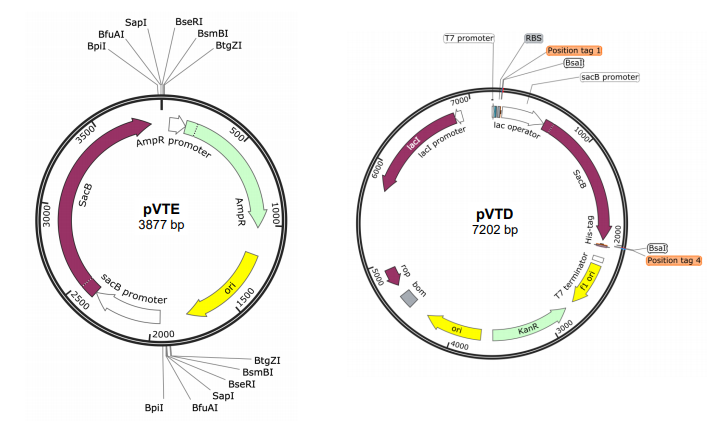


**Supplementary Figure 2.** **Map of the Versatile entry vector (pVTE) and destination vector (pVTD).** Used Tiles were cloned in the pVTE using typeIIs restriction enzymes and verified by sanger sequencing (Gerstmans et all. 2020). Full constructs containing the H-fiber, a linker, the EAD and a C-terminal his tag were subcloned in the pVTD2. This vector introduces a stop codon following the His-tag and enables expression of the chimeric proteins in *E. coli* using the T7 promotor and lac operon. The vector maps where made with SnappGene®, and the used protocols where taken from Gerstmans et al. (2020)

# 3 References

Gerstmans, H., Grimon, D., Gutiérrez, D., Lood, C., Rodríguez, A., van Noort, V., et al. (2020). A VersaTile-driven platform for rapid hit-to-lead development of engineered lysins. *Science Advances* 6(23)**,** eaaz1136. doi: 10.1126/sciadv.aaz1136.
