# Supplementary figures and images for: Developing Innolysins Against Campylobacter jejuni Using a Novel Prophage Receptor-Binding Protein
Source: Front Microbiol. 2021 Feb 1;12:619028. doi: 10.3389/fmicb.2021.619028 (PMC7882524; doi:10.3389/fmicb.2021.619028)

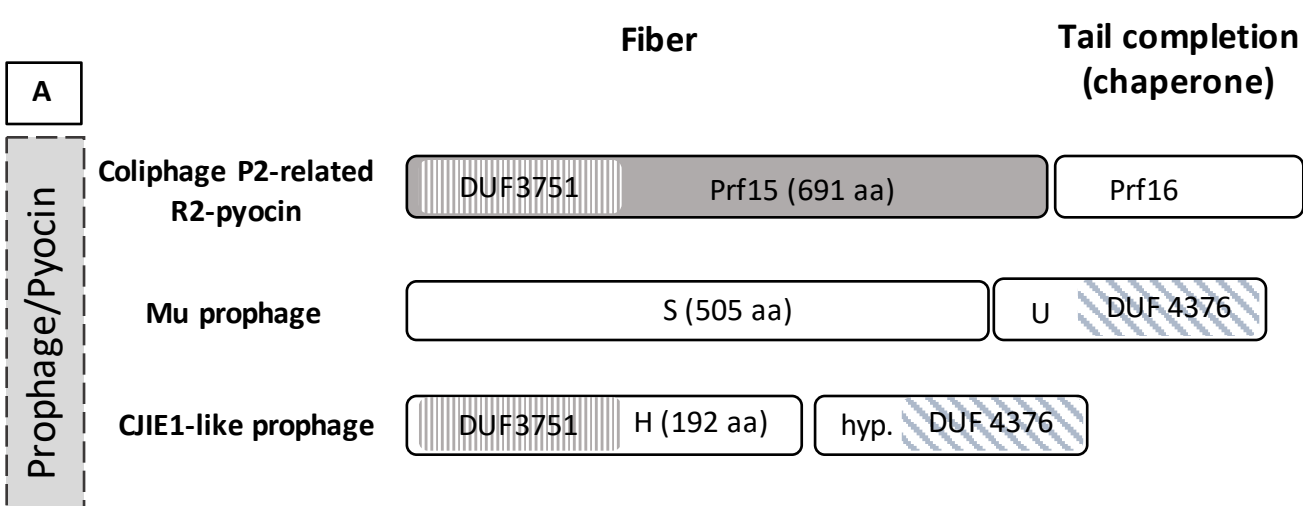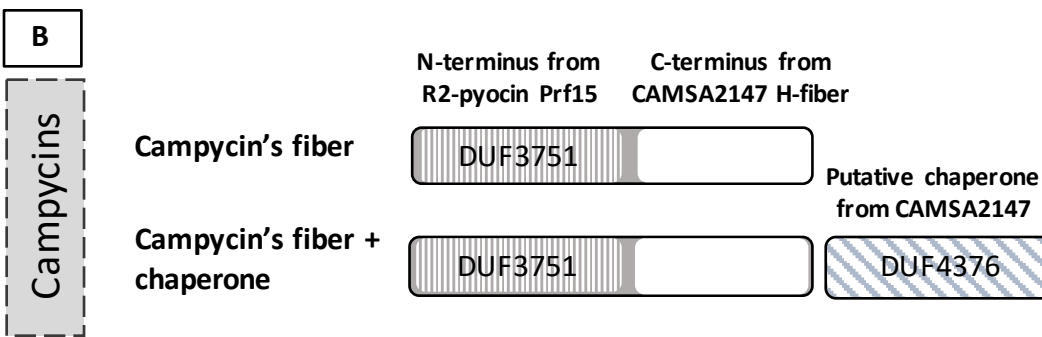

Supplement: Supplementary file 2 [file Data_Sheet_1.PDF]

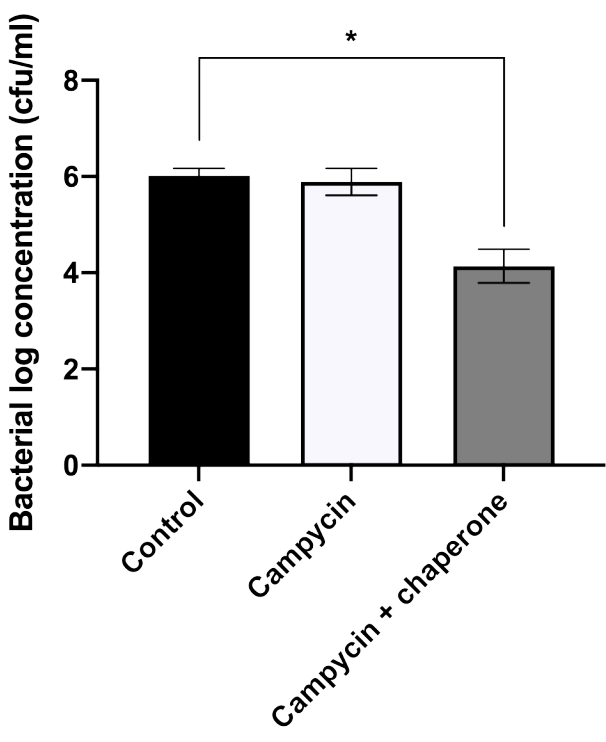

Supplement: Supplementary file 3 [file Data_Sheet_2.PDF]

**Innolysin Cj1:**

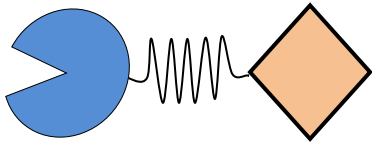

**Innolysin Cj2:**

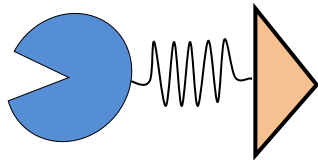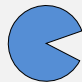

T5 endolysin

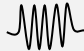

Linker

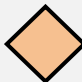

H-fiber

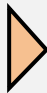

H-fiber part

Supplement: Supplementary file 4 [file Data_Sheet_3.PDF]

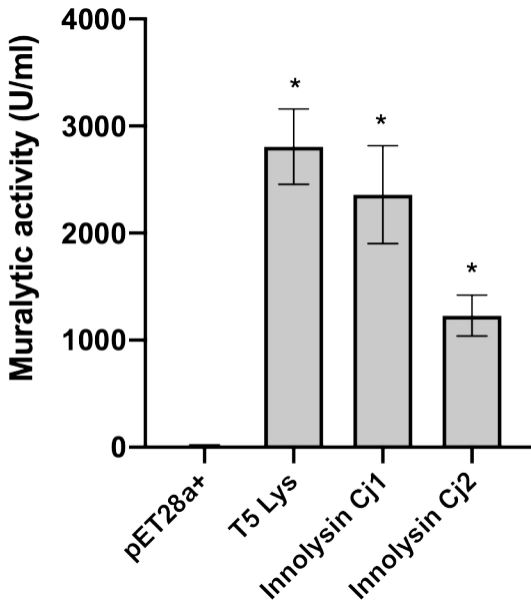

Supplement: Supplementary file 5 [file Data_Sheet_4.PDF]

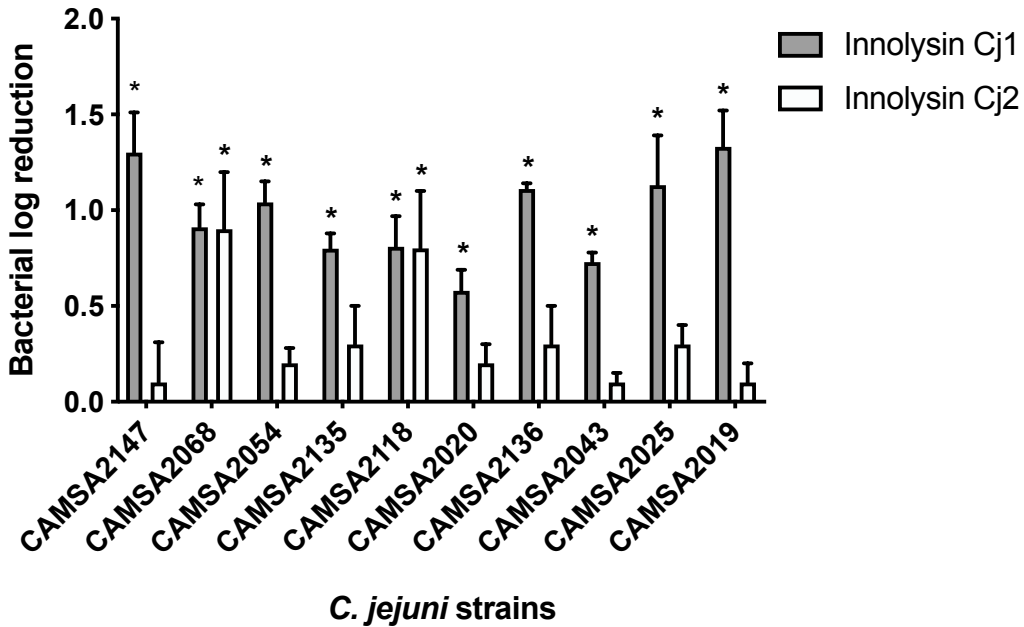

Supplement: Supplementary file 6 [file Data_Sheet_5.PDF]
